# Multi-Field Characterisation of Material Removal Processes in Ultrasonic Magnetorheological Chemical Compound Polishing of GaN Wafers

**DOI:** 10.3390/mi16050502

**Published:** 2025-04-25

**Authors:** Huazhuo Liang, Wenjie Chen, Youzhi Fu, Wenjie Zhou, Ling Mo, Qi Wen, Dawei Liu, Junfeng He

**Affiliations:** School of Mechatronic Engineering, Guangdong Polytechnic Normal University, Guangzhou 510665, China; lianghuazhuo@gpnu.edu.cn (H.L.); chenwenjie@gpnu.edu.cn (W.C.); yzfu@gpnu.edu.cn (Y.F.); zhouwj@gpnu.edu.cn (W.Z.); ling-mo@163.com (L.M.); scutwenqi@126.com (Q.W.); liudawei@gpnu.edu.cn (D.L.)

**Keywords:** GaN, ultrasonic magnetorheological chemical compound polishing, material removal, interactions

## Abstract

Gallium nitride (GaN), as the core material of third-generation semiconductors, has important applications in high-temperature, high-frequency, and high-power devices, but its polishing process faces many challenges. In this work, a multifield synergistic material removal model is established to study the material removal behaviour by ultrasonic magnetorheological chemical compound polishing (UMCP) of gallium nitride wafers, and the polishing processing under different polishing solution compositions and processing conditions is used to examine the effects of the ultrasonic, chemical, and mechanical effects on the material removal rate. The results show that mechanical removal dominates during UMCP, the chemical enhancement is slightly greater than the ultrasonic action, and the synergistic interaction between the range of factors promotes better removal of the GaN materials. The percentage of mechanical removal by abrasives is about 25% to 44.63%, the mechanical removal by magnetorheological effect polishing pads is about 14.66% to 23.94%, the removal due to chemical action is about 15.52% to 23.41%, the removal due to ultrasonic action is about 11.73% to 14.66%, and the percentage of interactive removal is 6.47% to 14.36%. The abrasive composition significantly enhances the mechanical removal effect, and a higher abrasive concentration correlates to a stronger mechanical removal effect. The concentration of hydrogen peroxide has a superior effect on the chemical reaction, and too high or too low a concentration of hydrogen peroxide weakens the chemical action effect. The results of the study can provide a basis for further research on the material removal mechanism of the GaN UMCP process.

## 1. Introduction

Gallium nitride is a third-generation semiconductor material and has high thermal conductivity, high breakdown field strength, high electron mobility, high radiation resistance, high temperature resistance, and long service life; thus, it is an important material and ideal substrate material for producing unmanned cars, wireless charging devices, photodetector devices, and 5G communication chips [1,2,3,4,5,6]. GaN substrate materials have stringent surface quality requirements, and minor surface defects and damage can severely affect the quality of the semiconductor devices [7,8]. However, due to the high hardness and chemical inertness of GaN materials, their processing is extremely difficult; this severely restricts their development and application [9,10].

Currently, the processing methods for GaN wafers are mainly traditional chemical–mechanical polishing (CMP) [11]. Aida et al. [12,13] used colloidal silica abrasives for 150 h of CMP processing of GaN and obtained an ultrasmooth surface without damage; however, the material removal rate (MRR) was only 17 nm/h. Although the process parameters were optimised systematically, the material removal rate did not exceed 0.1 μm/h. For this reason, many scholars have attempted to solve the problem of low processing efficiency by enhancing the oxidation properties of the polishing solution [14,15,16,17]. Murata et al. [18] used the hydroxyl radical generated from the Fenton reaction of hydrogen peroxide (H_2_O_2_) with divalent iron ions (Fe^2+^) to effectively soften GaN for the generation of Ga_2_O_3_ with a lower hardness. Wang et al. [19] used UV-catalysed decomposition of H_2_O_2_ to generate hydroxyl radicals to increase the material removal rate of the CMP process of GaN by 103 nm/h and obtained a smooth surface with a surface roughness Ra of 0.065 nm. Jiang et al. [20] used TiO_2_/ZrO_2_ soft and hard hybrid abrasives combined with photocatalytic assistance and the Fenton reaction to achieve high-efficiency and low-damage polishing of GaN, the material removal rate was 178.63 nm/h, and the surface roughness (Ra) was reduced to 0.244 nm. Pan et al. [21] used the electro-Fenton method to oxidise the GaN surfaces and to determine the removal mechanism of the oxide layer by studying the frictional wear behaviour and energy spectroscopy results. These results show that hydroxyl radicals could effectively oxidise and modify the GaN surface materials and that chemical–mechanical polishing using the Fenton reaction is more effective than polishing using conventional oxidants. The use of energy field-assisted methods could accelerate the generation of hydroxyl radicals, promote the progress of chemical reactions, and effectively improve the quality of material processing.

Combined with the low sensitivity of magnetorheological polishing to the hardness of abrasive grains [22,23,24], ultrasonic magnetorheological chemical compound polishing is proposed for the efficient and high-quality processing of GaN wafers using abrasive grains with high hardness. In previous studies, the reaction mechanism of the ultrasonic Fenton reaction on the GaN wafers was investigated, and a polishing process was carried out to obtain better surface quality. However, the ultrasonic magnetorheological chemical compound polishing material removal process is complex, and the material removal mechanism for the GaN substrates is not clear. In previous studies [25,26,27], the combined mechanical and chemical effects of the CMP process in materials such as SiC and diamond have been deeply analysed using molecular dynamics simulations, which help to understand the material removal mechanism of the CMP process in more depth.

There is a lack of systematic and in-depth research on the UMCP process of GaN substrates; especially, the mechanism of the influence of the interaction involving ultrasonic, chemical, and mechanical aspects on the removal of the material is not clear. Therefore, according to the effects of ultrasonic, chemical, and mechanical action, in this study, different polishing solutions and processing conditions were used for the polishing processing of the GaN wafers, and the effects of each factor on the material removal rate were investigated to analyse the material removal process under the action of multiple fields. The ultrasonic, chemical, and mechanical effects of the multi-field action of the composite machining process in material removal characteristics were further demonstrated through experiments.

## 2. Materials and Methods

### 2.1. Experimental Principles

The principle of the Fenton reaction for the oxidation of GaN is its ionisation using iron atoms under the weakly acidic action of hydrogen peroxide for the generation of Fe^2+^, as shown in Equation (1). Here, Fe^2+^ reacts with H_2_O_2_ in the Fenton reaction to produce the highly oxidising hydroxyl radical ·OH and forms Fe^3+^, as shown in Equation (2). Next, ·OH reacts with the GaN surface to form softer, easily removable Ga_2_O_3_, as shown in Equation (3). Moreover, the Fe^3+^ generated via Equation (2) reacts rapidly in the presence of H_2_O_2_ to form Fe^2+^ and H^+^ to promote ionisation (as shown in Equation (4)). During the above chemical reaction, Fe^2+^ does not disappear during the reaction process and only acts as a catalyst. When an ultrasonic field is incorporated into the Fenton reaction, on the one hand, the catalyst in the reaction is sufficiently dispersed through ultrasonic cavitation, and more reaction sites are generated [28,29]; on the other hand, the ultrasonic waves are able to form a localised region of high temperature and high pressure in the liquid phase [30]; this facilitates the redox reactions and accelerates the reaction rate. By introducing ultrasonic waves and the Fenton reaction with strong oxidative properties into the polishing process of the GaN wafers, the OH generated by the reaction oxidises the GaN surface to produce Ga_2_O_3_ with low hardness, thus attaining high-efficiency planarisation of the GaN wafers.

*Fe*^0^→*Fe*^2+^ + 2*e* (1)

(2) $$Fe^{2+}+H_{2}O_{2}\to Fe^{3+}+OH^{-}+\cdot OH$$

(3) $$GaN+6\cdot OH\to Ga_{2}O_{3}+H_{2}O+N_{2}↑$$

(4) $$Fe^{3+}+H_{2}O_{2}\to Fe^{2+}+\cdot OOH+H^{+}$$

**Figure 1:**
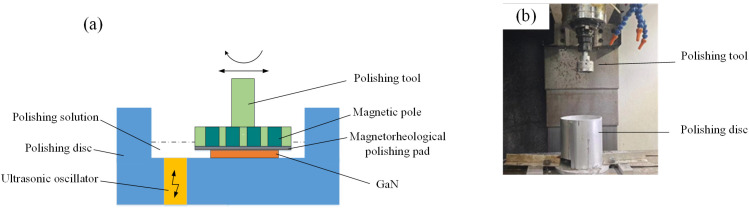
Polishing device, where (**a**) shows the processing principle, and (**b**) shows the device.

The polishing principle and device used in the experiment are shown in Figure 1a and Figure 1b, respectively; here, multiple magnetic poles are embedded within the polishing tool, and when the polishing tool approaches the GaN surface during polishing, the magnetic particles (carbonyl iron powder, CIP) in the polishing solution form a magnetorheological polishing pad under the action of the magnetic field; the magnetic field captures the abrasive particles and produces material removal behaviour on the GaN surface under the action of ultrasonic vibration.

### 2.2. Experimental Programme

The effect of ultrasonic magnetorheological chemical compound polishing of the GaN substrates is affected by ultrasonic, chemical, and mechanical effects, and the factors interact with each other. To study the effect of each factor of the polishing solution on the material removal rate, 11 groups of experiments were designed, and the experimental conditions and the composition of the polishing solution are shown in Table 1. Since the polishing solution in the experiments of groups 1, 2, and 3 had no magnetic particles, it could not form a magnetorheological polishing pad, and substantial material removal behaviour was difficult to induce. Therefore, the experiments of groups 1, 2, and 3 were carried out in the form of immersed GaN wafers to detect the surface conditions of the specimens. Polishing solutions of Exp. 4–Exp. 11 were used to polish the GaN wafer under the same process conditions to detect and analyse the polished surface roughness and material removal rate as a basis for studying the material removal of each factor. To examine the effect of the abrasive concentration on the material removal, all other conditions were kept constant and the abrasive concentration of the polishing solutions of Exps. 8, 9, 10, and 11 were set to 2 wt% and 4 wt% for repetitive polishing the GaN wafer. To investigate the oxidant concentration, all other conditions were kept constant, and the hydrogen peroxide content of the polishing solution was then changed to 1 wt% and 5 wt% for repetitive polishing, as described in Exps. 5, 7, 9, and 11, respectively. In the polishing experiment, the ultrasonic vibration frequency was 20 kHz, the power was 2 kW, the magnetic field strength in the polishing experiments was approximately 0.2 T, the workpiece speed was 350 r/min, and the polishing time was 1 h. The 4-inch single-crystal GaN was milled and cut as an experimental sample with a thickness of about 400 μm, roughness of about Ra 150 nm, and sample size of 1 cm × 1 cm. Fe_3_O_4_ particles were used as the catalysts, with a particle size of about 0.1 μm; carbonyl iron powder particles (CIP) were used as the magnetic particles, with a particle size of about 0.2 μm; and diamond particles were used as abrasive particles, with a particle size of about 0.1 μm. All experiments were conducted with Ga-polar as the machining surface for polishing.

Scanning electron microscopy (SEM, SU8220, form Hitachi High-Tech Corporation, Tokyo, Japan) was used to observe the oxidised surface and analyse the energy spectrum. The morphological characteristics and roughness values of the processed surface were observed via a white light interferometer (SuperViewW1, form ZYGO Corporation, Beijing, China), and the weight of the GaN wafer before and after polishing was detected using a precision electronic balance with a measurement accuracy of 0.1 mg (OHAUS-CP214, form OHAUS Corporation, Parsippany, NJ, USA). The material removal rate was subsequently calculated.

## 3. Experimental Results

### 3.1. Reaction Products on the GaN Surfaces

**Figure 2:**
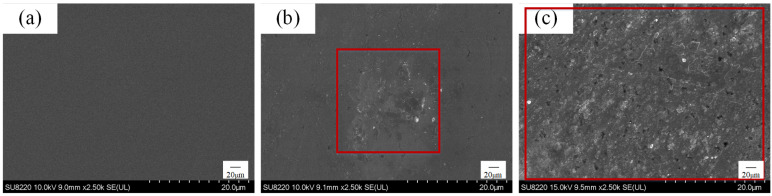
GaN surface case, where the (**a**–**c**) plots correspond to the results of Exps. 1, 2, and 3, respectively.

Exps. 1, 2, and 3 were carried out on GaN, and the GaN surface was observed after immersion for 1 h using SEM, and the results are shown in Figure 2. The GaN surface of Exp. 1 is flat and smooth, and no corrosion material is generated, as shown in Figure 2a. The GaN surface of Exp. 2 shows a localised corrosion phenomenon, as shown in Figure 2b, whereas a densely arranged scale-like corrosion layer is clearly observed on the GaN surface of Exp. 2, as shown in Figure 2c. Based on the energy spectrum results in Table 2, the GaN surface of Exp. 1 mainly contains two elements, Ga and N, with an atomic ratio of approximately 1:1, with a small amount of O (atomic percentage of 0.02%), and the appearance of oxygen is potentially due to the catalyst residue or contamination by other substances. The O content in Exp. 2 and Exp. 3 greatly increased, with atomic percentages of 13.61% and 23.26%, respectively, whereas the percentages of Ga and N in the two groups showed different degrees of increasing and decreasing changes, respectively. According to the reaction principle (see Equation (3)) and preliminary XPS tests [31], the surface corrosion layers of groups 2 and 3 are the reactants Ga_2_O_3_, and an escape of N occurs in the form of gasification during the reaction. These results indicate that the hydroxyl radicals generated by the reaction system can effectively react with the GaN surface to produce a softer Ga_2_O_3_ layer.

Measurements using a precision electronic balance revealed that the weights of the GaN substrates in Exps. 1, 2, and 3 before and after immersion remained almost unchanged; no material removal occurred from the GaN substrate in these three cases; here, Exp. 1 used only ultrasonic vibrations, Exp. 2 used only chemical reactions, and Exp. 3 used both ultrasonication and chemical action. The reaction produced Ga_2_O_3_, which increased the O content. The mass of the specimen was expected to have a slight increase; however, the decrease in the amount of N that escaped during the reaction resulted in almost no change in the mass of the specimen at the time of measurement.

### 3.2. Effects of the Different Polishing Solution Components on the Polishing Effect of GaN

**Figure 3:**
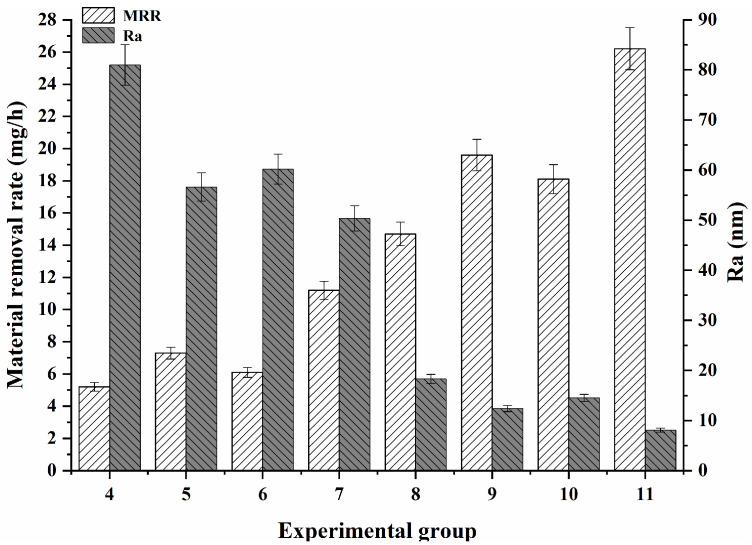
Polishing results of GaN for each group.

**Figure 4:**
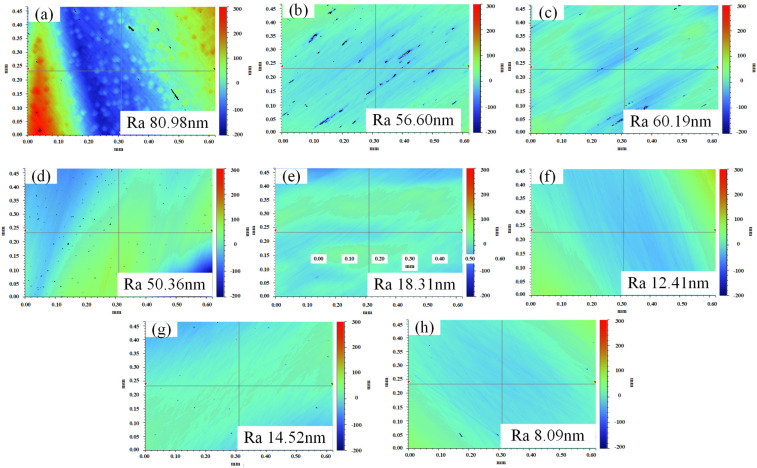
Surface morphology of each group after GaN processing, where the (**a**–**h**) plots correspond to the results of Exps. 4, 5, 6, 7, 8, 9, 10, and 11, respectively.

Exps. 4–11 were carried out on GaN, and the results are shown in Figure 3 and Figure 4. As shown in Figure 3, the material removal rate and surface roughness of each group greatly varied; here, the material removal rates of Exps. 4–11 were 4.5 mg/h, 7.3 mg/h, 6.1 mg/h, 11.2 mg/h, 14.7 mg/h, 19.6 mg/h, 18.1 mg/h, and 26.2 mg/h, and the surface roughness Ra values were 80.98 nm, 56.60 nm, 60.19 nm, 50.36 nm, 18.31 nm, 12.41 nm, 14.52 nm, and 8.09 nm, respectively. Due to the different experimental conditions of each group, they were difficult to analyse all together. Based on the experimental conditions, the results from Exps. 4–11 were examined in the three cases, with respect to the effects of abrasives, oxidisers, and ultrasonic fields.

Comparing the results from Exps. 4, 5, 6, and 7 with those from Exps. 8, 9, 10, and 11 to examine the effect of abrasives on processing, respectively, the abrasive addition resulted in a significant increase in the material removal rate of the latter and a significant decrease in the surface roughness values. From the surface images, the following observations were made: the GaN surface area of Exp. 4 greatly varied in height, with a rougher surface and evident pits (see Figure 4a); a small number of pits were observed on the surface of Exps. 5, 6, and 7, and the overall machined surface was flatter (see Figure 4b–d); and the pits disappeared from the machined surfaces of Exps. 8, 9, 10, and 11, with a significant improvement in surface quality (see Figure 4e–h); these results indicated that only carbonyl iron powder formed a flexible magnetorheological polishing pad with a weak removal ability for the GaN material and that the abrasive had a greater enhancement effect on the material removal ability.

By examining the role of the oxidising agent, the results from Exps. 4, 6, 8, 10 and Exps. 5, 7, 9, and 11 are compared accordingly. The material removal rate and surface quality of the latter are slightly better than those of the former; these results, combined with those from the immersion reaction in Section 3.1 and the surface morphology in Figure 4, indicate that the chemical action prompts the formation of an oxide layer that is softer on the GaN surface and favourable for the processing behaviour. Based on a comparison of Exps. 4, 5, 8, and 9 and Exps. 6, 7, 10, and 11, respectively, for examining the effect of ultrasonication on processing, ultrasonication promotes the processing behaviour.

### 3.3. Effect of the Abrasive Concentration on the GaN Material Removal

**Figure 5:**
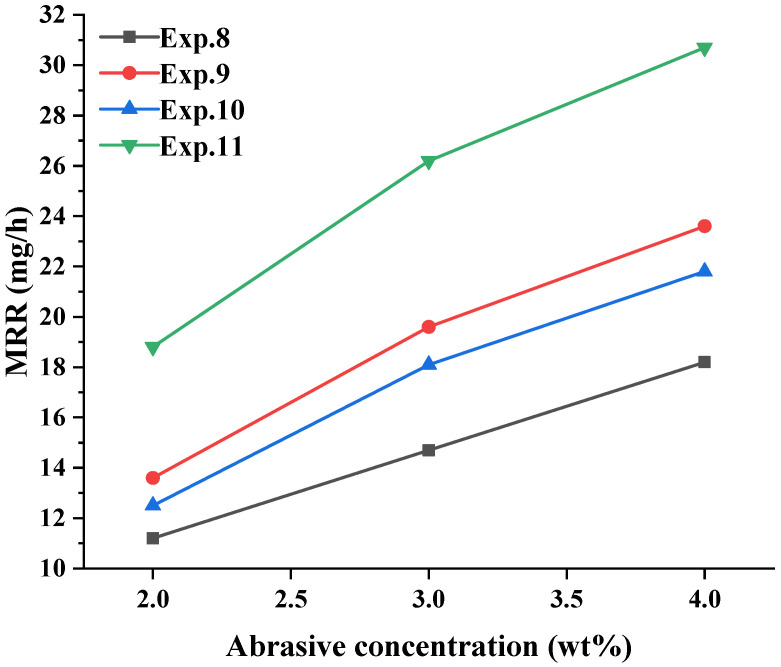
Effects of different abrasive concentrations on the material removal rate.

Abrasiveness has a major influence on GaN material removal; the abrasive concentration was changed from 3 wt% to 2 wt% and 4 wt% for Exps. 8, 9, 10, and 11, the other conditions in the polishing experiments were kept constant, and the results from the material removal rate were obtained, as shown in Figure 5.

As shown in Figure 5, the material removal rates of these four groups of Exps. 8, 9, 10, and 11 increased with increasing abrasive concentration; these results indicated that the enhancement of the mechanical removal effect was clearly caused by the abrasive component. Under the condition of the same abrasive concentration, the results from the material removal rate were Exp. 11 > Exp. 9 > Exp. 10 > Exp. 8. Compared to Exp. 8, Exp. 10 showed an increase in the ultrasonic effect, and ultrasonic vibration increased the friction and impact between the GaN surface and polishing tool to enhance the removal effect. Compared with Exp. 8, Exp. 9 added an oxidant; thus, the surface modification behaviour of the chemical reaction promoted material removal. The results from this sequence also showed that the effect of chemical action on the material removal behaviour was greater than that of ultrasonic action. Exp. 11 introduced both chemical action and ultrasonic action, and its material removal rate was the highest, mainly because ultrasonic action could promote the chemical reaction to cause the ultrasonic Fenton effect, and at the same time, ultrasonic high-frequency vibration promoted the abrasive and carbonyl iron powder particles in the polishing fluid to produce the high-frequency impacts on the GaN surface [32,33], which enhances the mechanical removal ability of the abrasive and polishing pads.

### 3.4. Effects of the Hydrogen Peroxide Concentration on the Polishing Effect of GaN

**Figure 6:**
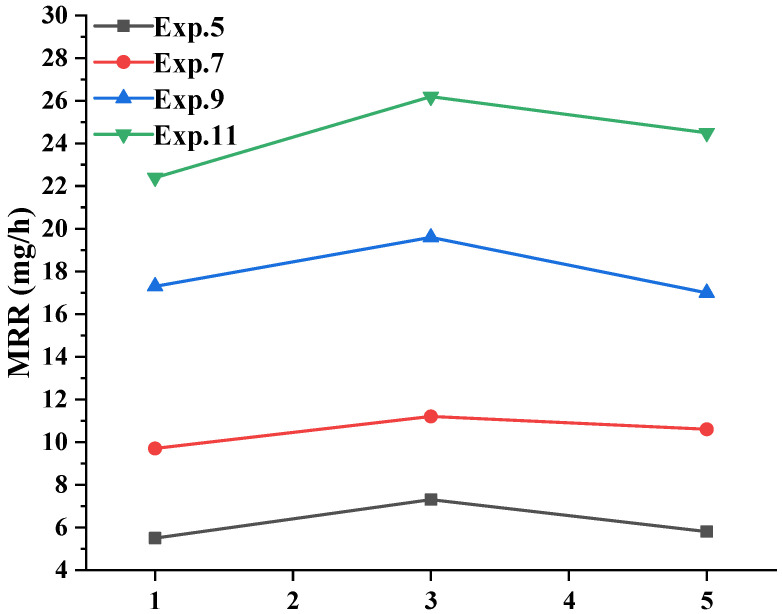
Effects of the different oxidant concentrations on the material removal rates.

For Exps. 5, 7, 9, and 11, the concentration of hydrogen peroxide in the polishing solution was changed to 1 wt% and 5 wt% for the polishing experiments, and the results of the material removal rates are shown in Figure 6. As shown in Figure 6, the material removal rates of all four groups initially increased and then decreased with increasing H_2_O_2_ concentration. Under the same H_2_O_2_ concentration conditions, the material removal rates were always Exp. 11 > Exp. 9 > Exp. 7 > Exp. 5, and the results from Exp. 11 and Exp. 9 were approximately one time greater than those from Exp. 7 and Exp. 5.

Compared with Exp. 5, Exp. 7 showed an increase in the ultrasonic action, and Exp. 9 showed an increase in the abrasive component, whereas Exp. 11 showed increases in both. The enhancement of material removal behaviour by ultrasonication was limited, whereas the enhancement by the abrasive composition was significant, and a clear interaction was observed between ultrasonication and abrasives. The results from the material removal rate curve changed because increasing the concentration of H_2_O_2_ tended to produce a quenching reaction, capturing emerging hydroxyl radicals [34]; this led to a weakening of the oxidation reaction on the surface of GaN, resulting in a decrease in the material removal rate.

## 4. Analysis and Discussion

### 4.1. Material Removal Composition for Compound Polishing

The effect of ultrasonic magnetorheological chemical compound polishing of GaN is influenced by ultrasonic, chemical, and mechanical actions, and these factors interact with each other. The results from the above polishing experiments qualitatively reveal that the abrasive component on the material removal in the ultrasonic magnetorheological chemical compound polishing of GaN has the greatest effect, and the ultrasonic and chemical effects have a certain promotion effect on material removal. A similar approach to the factor effects of multi-field composite processing has been reported [35,36], and drawing on that approach, the following analysis is made: To better analyse the synergistic effect of ultrasonic magnetorheological chemical polishing of GaN, the total amount of GaN removal is divided into the material removal caused by a single factor and the amount of material removal caused by a multifactor, as shown in Equation (5). Among them, the material removal amount caused by a single factor is the removal amount by mechanical action, ultrasonic action, and chemical action; the material removal amount caused by multiple factors includes the material removal amount by two interactions and three interactions between mechanical, ultrasonic, and chemical action.

(5) $$M_{total}=M_{m}+M_{u}+M_{c}+M_{m-u}+M_{m-c}+M_{u-c}+M_{m-u-c}$$

where M_total_ represents the total material removal; M_m_, M_u_, and M_c_ represent material removal under mechanical, ultrasonic, and chemical effects, respectively; M_m-u_, M_m-c_, and M_u-c_ represent material removal under the synergistic interactions of mechanical and ultrasonic, mechanical and chemical, and ultrasonic and chemical effects, respectively; and M_m-c-u_ represents material removal under the interactions of mechanical, chemical, and ultrasonic effects.

In ultrasonic magnetorheological chemical compound polishing, the mechanical material removal is mainly composed of both the abrasive and polishing-pad removal, and the abrasive component cannot act separately from the polishing pad; thus, disregarding all the interactions between the abrasive component and the polishing pad, i.e., the total amount of material removal, M_total_, can be further expressed as Equation (6):

(6) $$M_{total}=M_{a}+M_{p}+M_{u}+M_{c}+M_{a-u}+M_{a-c}+M_{p-u}+M_{p-c}+M_{u-c}+M_{a-u-c}+M_{p-u-c}$$

where M_a_ and M_p_ are the materials removed by the action of abrasives and polishing pads, respectively, and M_a-c-u_ and M_p-c-u_ are the materials removed by the interaction of abrasives and polishing pads with chemistry and ultrasound, respectively.

According to the polishing-solution composition in Table 1, the material removal rate of Exp. 11 is used as the total material removal rate of the compound polishing M_total_, and the material removal role of each factor in the GaN-polishing process is further quantitatively analysed. Since only ultrasonication is added to Exp. 1, its material removal can be considered as M_u_; Exp. 2 has only chemical components, and its material removal is M_c_; Exp. 3 adds ultrasonication on the basis of Exp. 2, and its material removal is determined by the chemical, ultrasonication, and the interaction of the two (M_c_, M_u_, and M_u-c_); in Exp. 4, only carbonyl iron powder is added to the polishing solution, and its material removal is entirely generated by the mechanical removal of M_p_ from the formed flexible polishing pads; in Exp. 5, hydrogen peroxide and carbonyl iron powder are added, and its material removal consists of M_p_, M_c_, and M_p-c_; in Exp. 6, ultrasonic assistance is added based on Exp. 4, and its material removal consists of M_u_, M_p_, and M_p-u_; Exp. 7 adds hydrogen peroxide based on Exp. 6, its material removal from the polishing process is added to the chemical removal M_c_, the interaction between chemical and ultrasound M_u-c_, the interaction between chemical and polishing pads, and the interaction of all three together is M_p-u-c_; and similarly, Exp. 8, Exp. 9, Exp. 10, and Exp. 11 add abrasive components to Exp. 4, Exp. 5, Exp. 6, and Exp. 7, respectively, and their material removal is increased by the abrasive removal M_a_ and the corresponding interaction removal effect. The material removal for the specific groups of experiments can be expressed as Equations (7)–(17):

(7) $$M_{Exp.1}=M_{u}$$

(8) $$M_{Exp.2}=M_{c}$$

(9) $$M_{Exp.3}=M_{u}+M_{c}+M_{u-c}$$

(10) $$M_{Exp.4}=M_{p}$$

(11) $$M_{Exp.5}=M_{p}+M_{c}+M_{p-c}$$

(12) $$M_{Exp.6}=M_{p}+M_{u}+M_{p-u}$$

(13) $$M_{Exp.7}=M_{p}+M_{u}+M_{c}+M_{p-u}+M_{p-c}+M_{u-c}+M_{p-u-c}$$

(14) $$M_{Exp.8}=M_{a}+M_{p}$$

(15) $$M_{Exp.9}=M_{a}+M_{p}+M_{c}+M_{a-c}+M_{p-c}$$

(16) $$M_{Exp.10}=M_{a}+M_{p}+M_{u}+M_{a-u}+M_{p-u}$$

(17) $$M_{Exp.11}=M_{total}$$

Based on the experimental results from Section 3.1 and the relationships in Equations (7)–(17), the relationship between the material removal rates due to each factor is as follows:

(18) $$M_{u}=M_{Exp.1}\approx 0$$

(19) $$M_{c}=M_{Exp.2}\approx 0$$

(20) $$M_{u-c}=M_{Exp.3}-M_{Exp.1}-M_{Exp.2}\approx 0$$

(21) $$M_{p}=M_{Exp.4}$$

(22) $$M_{a}=M_{Exp.8}-M_{Exp.4}$$

(23) $$M_{p-c}=M_{Exp.5}-M_{Exp.2}-M_{Exp.4}$$

(24) $$M_{p-u}=M_{Exp.6}-M_{Exp.1}-M_{Exp.4}$$

(25) $$M_{a-c}=M_{Exp.9}-M_{Exp.5}-M_{Exp.8}+M_{Exp.4}$$

(26) $$M_{a-u}=M_{Exp.10}-M_{Exp.6}-M_{Exp.8}+M_{Exp.4}$$

(27) $$M_{p-u-c}=M_{Exp.7}-M_{Exp.5}-M_{Exp.6}+M_{Exp.4}$$

(28) $$M_{a-u-c}=M_{Exp.11}-M_{Exp.4}-M_{Exp.7}-M_{Exp.9}-M_{Exp.10}+M_{Exp.5}+M_{Exp.6}+M_{Exp.8}$$

### 4.2. Analysis of the Role of Factors in Material Removal

**Figure 7:**
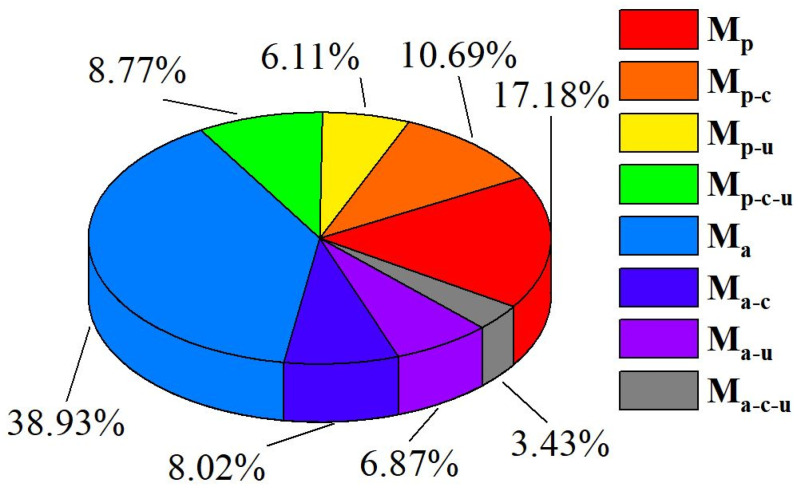
Contribution of material removal by factor.

According to Equations (18)–(28) and based on the analysis of the polishing results from Section 3.2, the contribution of the material removal of each factor to the total removal during ultrasonic magnetorheological chemical compound polishing can be obtained. The material removal result of Exp. 11 was used as Mtotal, which corresponds to the material removal rates of Exps 1–10 under the same process conditions, in order to calculate the removal rate of each factor (calculated according to Equations (18)–(28)), and thus the contribution of each component to the total removal rate. As shown in Figure 7, the abrasives constitute the largest proportion of material removal for GaN, and M_a_ reaches 38.93%, followed by the proportion of polishing-pad removal, M_p_, of 17.18%; both of these belong to the category of mechanical removal, and the remaining portion comes from the removal caused by the interaction between multiple factors. Since chemical action induces oxidative modification of the GaN surface, and the product Ga_2_O_3_ is more susceptible to mechanical removal, both M_p-c_ and M_a-c_ need to be considered chemical-induced material removal. Similarly, ultrasonication is effective in enhancing mechanical removal, and M_p-u_ and M _a-u_ are considered ultrasonication-induced material removal. On the other hand, ultrasonication enhances the Fenton reaction and thus the mechanical removal; therefore, M_p-u-c_ and M_a-u-c_ need to be considered synergistic interactions among ultrasonication and chemical and mechanical removal. According to these analyses, the percentage of material removal due to chemical action is 18.71%, the percentage of material removal due to ultrasonic action is 12.98%, and the percentage of interactive removal is 12.20%. Based on these results, mechanical removal is dominant in ultrasonic magnetorheological chemical compound polishing (the sum of M_a_ and M_p_ is 56.11%), the chemical enhancement is slightly greater than the ultrasonic action, and the synergistic interaction between various factors promotes an improvement in the material removal of GaN.

**Figure 8:**
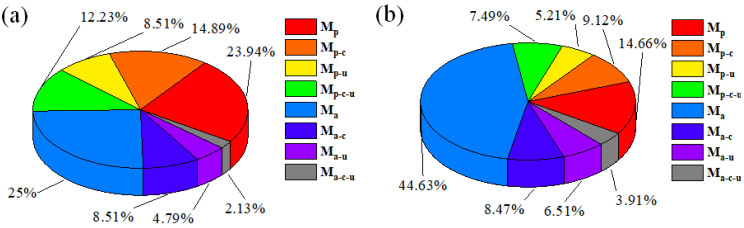
Material removal contribution of each factor at different abrasive concentrations: (**a**) 2 wt% and (**b**) 4 wt%.

To further analyse the effects of the changes in the abrasive concentration on the material removal contribution, the results from Section 3.3 were analysed, and the results are shown in Figure 8. Based on the results from Figure 5 and Figure 7 with an abrasive concentration of 3 wt%, the abrasive has the greatest influence on the material removal of GaN compound polishing, and when the abrasive concentration of 2 wt% and 4 wt%, the proportion of M_a_ is 25% and 44.63%, respectively. With the increasing abrasive concentration, the proportion of material removal from the flexible polishing pads due to magnetic particles M_p_ decreased from 23.94% to 14.66%. Similarly, the proportion of material removal due to ultrasonics, chemistry, and the interaction of the three components also decrease to varying degrees. The percentage of material removal due to chemical action decreased from 23.41% to 17.59%, the percentage of material removal due to ultrasound action decreased from 13.30% to 11.73%, and the percentage of interaction removal decreased from 14.36% to 11.40%. This occurs because the increase in the abrasive concentration leads to an increase in the total amount of material removed M_total_, whereas the polishing-pad removal remains constant and results in a decrease in the relative proportion of material removed M_p_. Similarly, the proportions of ultrasonic, chemical, and triple interactions also decrease.

As the abrasive concentration increases, the amount of abrasive involved in effective removal behaviour increases, thus increasing the mechanical removal capacity. Notably, even at low abrasive concentrations, the percentage of mechanical removal is still greater than the percentage of material removal due to chemical action. Based on these results, the generation rate of the GaN surface oxide layer during processing is slower than its removal rate, and the abrasive component greatly affects a fresh, unoxidised GaN surface.

**Figure 9:**
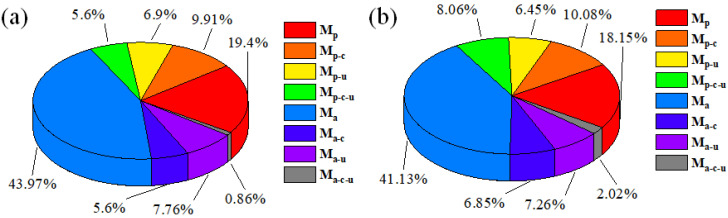
Material removal contribution of each factor using different hydrogen peroxide concentrations: (**a**) 1 wt% and (**b**) 5 wt%.

The results from Section 3.4 were analysed to obtain the material removal contribution of each factor at different hydrogen peroxide concentrations, as shown in Figure 9. Compared with Figure 7 at a hydrogen peroxide concentration of 3 wt%, the sum of the proportions of the material removal caused by the abrasives and polishing pads was always the largest with increasing hydrogen peroxide concentration; although different degrees of change were observed. The corresponding chemical-induced material removal percentages (sum of M_p-c_ and M_a-c_) were 15.51%, 18.71%, and 16.93%, respectively. The same pattern was observed for ultrasonication and the three interactions; and the proportions reached maximum values when the hydrogen peroxide concentration was 3 wt%, and a decrease in the proportions was observed when either the hydrogen peroxide concentration was increased or decreased. These results indicated that the hydrogen peroxide concentration has a superior effect on the chemical reaction effect and that too high or too low a concentration of hydrogen peroxide could weaken the effect of its chemical action instead.

### 4.3. Analysis of the Material Removal Behaviour for Compound Polishing

The proportion of mechanical removal in ultrasonic magnetorheological chemical compound polishing of GaN is the largest; here, the removal ability of the abrasive is much greater than that of the polishing pads, the proportion of material caused by ultrasonic, chemical, and interaction does not differ much, and all interactions have a certain auxiliary effect on the removal of the material. Therefore, the removal of material from the GaN surface is considered to occur mainly by scratching the material with abrasive grains. In contrast to the chemical–mechanical polishing of silicon wafers [35], whose material removal behaviour is derived mainly from the mechanochemical interactions between the abrasive and the polishing solution, very little removal is achieved by abrasives or chemical action alone. This occurs because the structural differences in the crystals causes difficulty in the GaN oxidation modification. Second, to ensure the quality of the processed surface, the CMP process is carried out with a very low hardness SiO_2_ abrasive; however, ultrasonic magnetorheological chemical compound polishing is carried out with a flexible polishing pad formed by the carbonyl iron powder under a magnetic field, which can adapt to high-hardness abrasive grains. The abrasive removal capacity of the process using diamond abrasives is much greater than that of the CMP system polishing process.

Ultrasonic magnetorheological chemical compound polishing combines the advantages of ultrasonic action, magnetorheological polishing, and chemical–mechanical polishing; through a chemical reaction, a chemical reaction layer is first formed on the surface of the workpiece, and then the reaction layer is removed by the mechanical action of the magnetorheological effect polishing pads and abrasive grits. The principle involves the use of abrasives, magnetic particles (CIPs), oxidants (H_2_O_2_), and catalysts (Fe_3_O_4_) formulated into a magnetorheological chemical-polishing solution. The magnetic particles in the polishing solution under the action of the magnetic field form a magnetic chain structure, and the abrasive on the magnetic chain structure of the string structure is clamped to the surface extrusion of the workpiece. At the same time, the oxidising agent produces a strong oxidising hydroxyl radical to oxidise the surface material of GaN, and the oxidised layer generated is more easily removed by the abrasives and the polishing pad. On this basis, the introduction of ultrasonic vibration, on the one hand, can improve the kinetic energy of solid particles and increase the friction and impact between the workpiece surface and the polishing tool; on the other hand, it can also promote chemical reactions for the highly efficient removal of the surface material. Ultrasonic magnetorheological chemical compound polishing integrates ultrasonic action, magnetorheological polishing, and chemical–mechanical polishing features. The material removal process is complex, and many factors affect the need for appropriate control of the factor variables to coordinate the ultrasonic, chemical, and mechanical action to obtain high-efficiency and high-quality polished surfaces.

## 5. Conclusions

(1) Mechanical removal dominates ultrasonic magnetorheological chemical compound polishing, chemical enhancement is slightly greater than ultrasonic action, and synergistic interactions between the range of factors promote better removal of the GaN materials.

(2) The abrasive composition significantly enhances the mechanical removal effect, and a higher abrasive concentration correlates to a stronger mechanical removal effect. The concentration of hydrogen peroxide has a superior effect on the chemical reaction, and too high or too low a concentration of hydrogen peroxide weakens the effect of chemical action.

(3) The material removal process of ultrasonic magnetorheological–chemical composite polishing is complex and requires proper control of the factor variables to coordinate ultrasonic, chemical, and mechanical actions for the attainment of an efficient and high-quality polished surface.

## Figures and Tables

**Table 1:** Experimental conditions and the composition of the polishing solution.

No.	Oxidant (H_2_O_2_)	Catalyst (Fe_3_O_4_)	Magnetic Oarticle (CIP)	Abrasive	Ultrasonic
1	-	2 wt%	-	-	+
2	3 wt%	2 wt%	-	-	-
3	3 wt%	2 wt%	-	-	+
4	-	2 wt%	12 wt%	-	-
5	3 wt% (1 wt%, 5 wt%)	2 wt%	12 wt%	-	-
6	-	2 wt%	12 wt%	-	+
7	3 wt% (1 wt%, 5 wt%)	2 wt%	12 wt%	-	+
8	-	2 wt%	12 wt%	3 wt% (2 wt%, 4 wt%)	-
9	3 wt% (1 wt%, 5 wt%)	2 wt%	12 wt%	3 wt% (2 wt%, 4 wt%)	-
10	-	2 wt%	12 wt%	3 wt% (2 wt%, 4 wt%)	+
11	3 wt% (1 wt%, 5 wt%)	2 wt%	12 wt%	3 wt% (2 wt%, 4 wt%)	+

**Table 2:** GaN surface element composition.

Exp. 1			Exp. 2			Exp. 3		
Elt.	Mass Norm.	Atom	Elt.	Mass Norm.	Atom	Elt.	Mass Norm.	Atom
O	0.04%	0.02%	O	4.51%	13.61%	O	8.37%	23.26%
N	16.29%	49.22%	N	7.28%	25.17%	N	7.04%	22.45%
Ga	83.67%	50.76%	Ga	88.21%	61.22%	Ga	84.59%	54.19%
Total	100%	100%	Total	100%	100%	Total	100%	100%

## Data Availability

The original contributions presented in this study are included in the article. Further inquiries can be directed to the corresponding author.
